# 

**Published:** 1894-06-30

**Authors:** 


**Notices of Births, Deaths, and Marriages** are inserted in this column at a charge of 2s. 6d. for an announcement not exceeding four lines.

**Notice to Advertisers and Subscribers.**

All Advertisements, Orders for Copies of The Hospital, and Business Communications should be addressed to **The Publisher, NOT TO THE EDITOR,** The Hospital, 428, Strand, W.C.

The Cost of Subscription, post free, is as follows:—Per week, 2½d.; per quarter, 2s. 8d.; per half-year, 5s. 3d.; per annum, 10s. 6d. Foreign:—Per Annum, 12s. 8d.; payable, in all cases, in advance.

### NOTICE TO CORRESPONDENTS.

All MS., Letters, Books for Review, and other matters intended for the Editor should be addressed The Editor, The Lodge, Porchester Square, London, W.

The Editor cannot undertake to return rejected MS., even when accompanied by stamped directed envelope.

**"Burdett's Hospital Annual," 1894.**

Fifth year of publication. 900 pp. Boards, gilt lettering. A most complete guide to British, Colonial, Indian, and American Hospitals, Dispensaries, Nursing and Convalescent Institutions, and Asylums. Price 5s. Published by The Scientific Press (Limited), 428, Strand, W.C.

**NOTICE.—The Index for Vol. XV. (ending March 31st, 1894) is on sale at the Office of this Journal, price 2½d., post free.**

### THE STUDENT OF MEDICINE.

### APPOINTMENTS.

Mr. J. Arlidge, appointed Honorary Assistant Ophthalmic Surgeon to the North Staffordshire Infirmary. C. P. Bullock, M.A.Oxon, L.R.C.P.Lond., M.R.C.S.Eng., appointed Deputy Coroner for Oswestry. T. K. Dalziel, M.B., C.M.Edin., F.F.P.S.Glasg., appointed Honorary Surgeon to the Glasgow Royal Hospital for Sick Children. W. Deans, M.B., C.M.Aberd., reappointed Medical Officer of Health to the Ramsbottom Local Board. J. W. Dickson, M.B., B.C.Cantab., appointed Anæsthetist to the Grosvenor Hospital for Women and Children, Vincent Square, S.W. G. W. Eglington, L.R.C.P.Edin., L.F.P.S.Glasg., reappointed Medical Officer of Health to the Street Local Board. A. K. Gale, L.R.C.P.Lond., M.R.C.S.Eng., reappointed Medical Officer of Health to the Ecclesall Bierlow Rural Sanitary Authority. W. C. Hamilton, M.B. C.M.Edin., appointed Junior House Surgeon to the Blackburn and East Lancashire Infirmary. M. F. Hopson, L.D.S.Eng., appointed Dental Surgeon to the Hampstead Hospital. A. F. Keyworth, M.R.C.S., L.R.C.P.I., appointed Certifying Factory Surgeon for the Marple District. A. W. B. Livesay, M.B., C.M.Edin., appointed House Surgeon to the Royal Victoria Hospital, Bournemouth. C. E. Maude, M.B., C.M.Edin., appointed Senior House Surgeon to the Blackburn and East Lancashire Infirmary. J. M. Nicoll, M.B., C.M.Edin., appointed Medical Officer of Health for the Borough of Jarrow, and Medical Superintendent to the Borough Hospital. Mr. E. W. Parsey, appointed Assistant Medical Officer of the Workhouse and Infirmary of the Parish of Paddington. H. Phillpotts, M.D., L.R.C.P.Lond., M.R.C.S.Eng., appointed Surgeon to the Ealing Cottage Hospital. D. W. Robertson, L.R.C.P.Edin., L.M., M.R.C.S.Eng., reappointed Medical Officer of Health to the Pickering Local Board. A. W. Sheen, M.D., B.S.Lond., F.R.C.S.Eng., appointed Senior Resident Medical Officer to the Glamorgan and Monmouthshire Infirmary, Cardiff. F. S. Toogood, M.D.Lond., M.R.C.S.Eng., appointed Medical Superintendent of the Infirmary of the Lewisham Union. W. H. Webb, M.D.Durh., L.R.C.P.Lond., M.R.C.S.Eng., appointed Medical Officer of Health to the Kingsbridge Rural Sanitary Authority.

### VACANCIES.

### Resident Medical Officers.

Brighton, Hove, and Preston Dispensary.—House Surgeon for the parent establishment. Salary £140 per annum, with furnished apartments, coal, gas, and attendance, but no board. Applications and testimonials to J. W. Stride, Assistant Secretary, before July 10th.

County Asylum, Rainhill, near Liverpool.—Assistant Medical Officer, to act as *Locum Tenens* for about two months. Salary £2 2s. per week, with board, lodging, &c. Applications to the Medical Superintendent.

Croydon General Hospital.—House Surgeon. Appointment for two years. Salary £100 per annum, increasing £10 per annum up to £120. Applications and testimonials to the Secretary, J. Jones, by July 6th.

St. Luke's Hospital, London, E.C.—Clinical Assistant. Appointment for six months. Board and residence provided. Applications to Percy de Bathe, M.A., Secretary.

Sunderland Borough Lunatic Asylum.—Medical Superintendent, doubly qualified. Salary £350 per annum, with furnished house, board for self and wife (if married), washing, coals, light, two servants, and use of garden. Applications, endorsed "Medical Superintendent," to Fras. M. Bowey, Clerk to the Visiting Committee, Town Hall, Sunderland, by June 30th.

West Norfolk Hospital, King's Lynn.—House Surgeon, who will also act as Secretary to the Weekly Board. Salary £80, rising £10 annually to £100, with board, residence, and washing. Applications and copies of testimonials to S. R. Lister, Secretary, by July 7th.

### Non-Resident Medical Officers.

Royal London Ophthalmic Hospital, Moorfields.—Curator, non-resident. Appointment for one year; renewable. Salary £120 per annum. Applications to the Secretary by June 30th.

Salters' Company.—Research Fellowship in Experimental Pharmacology. Annual value of £100, and is tenable in the Medical School of St. Thomas's Hospital. Applications to the Secretary to the Medical School, St. Thomas's Hospital, S.E., before June 30th.

Salters' Company.—Research Fellowship in Chemistry. Annual value of £100, tenable in the Research Laboratory of the Pharmaceutical Society. Applications to Professor Dunstan, F.R.S., Director of the Research Laboratory of the Pharmaceutical Society, 17, Bloomsbury Square, W.C., before June 30th.

University College, Dundee, St. Andrews University.—Professor of Chemistry. Applications to R. N. Kerr, Secretary, by July 7th.

University of Aberdeen.—Examiners in Medicine. Applications and testimonials to Robert Walker, Secretary of the University Court, on or before July 4th.

University of Edinburgh.—Chemical Assistant to Professor of Physiology. Salary £180 per annum. Applications to the Secretary of the University Court before July 1st.

Westminster Hospital Medical School.—Demonstrator of Anatomy. Applications to Mr. Spencer, the Dean, before July 10th.

### PASS LIST.

### ROYAL COLLEGE OF SURGEONS OF ENGLAND.

The following gentlemen were, at the ordinary meeting of the Council on Thursday, June 14th, elected examiners for the ensuing year in the subjects indicated: *First Examination.*—Elementary Anatomy: L. A. Dunn, M.B., B.S.Lond., F.R.C.S.Eng., Guy's Hospital; W. H. H.

THE STUDENT OF MEDICINE—*continued.*

Jessop, M.B.Cantab., F.R.C.S.Eng., St. Bartholomew's Hospital; J. E. Lane, F.R.C.S.Eng., St. Mary's Hospital; H. F. Waterhouse, M.D., C.M.Edin., F.R.C.S.Eng., Charing Cross Hospital. Elementary Physiology: J. R. Bradford, M.D., D.Sc.Lond., M.R.C.S.Eng., University College Hospital. Elementary Biology: H. P. Dean, M.B., B.S.Lond., F.R.C.S.Eng., London Hospital; T. W. Shore, M.D., B.Sc.Lond., M.R.C.S.Eng., St. Bartholomew's Hospital. *Second Examination.*—Anatomy: R. C. Lucas, M.B., B.S.Lond., F.R.C.S.Eng., Guy's Hospital; G. H. Makins, F.R.C.S.Eng., St. Thomas's Hospital; W. J. Walsham, F.R.C.S.Eng., St. Bartholomew's Hospital; A. H. Young, M.B., C.M.Edin., F.R.C.S.Eng., Owens College, Manchester. Physiology: W. D. Halliburton, M.D., B.Sc.Lond., M.R.C.S.Eng., King's College Hospital; D'A. Power, M.B.Oxon., F.R.C.S.Eng., St. Bartholomew's; W. G. Spencer, M.B., M.S.Lond., F.R.C.S.Eng., Westminister Hospital. *First Professional Examination for the Fellowship*—Anatomy: W. Anderson, F.R.C.S.Eng., St. Thomas's Hospital; W. F. Haslam, F.R.C.S.Eng., Mason College, Birmingham; W. H. A. Jacobson, M.B., M.Ch.Oxon., F.R.C.S.Eng., Guy's Hospital; C. B. Lockwood, F.R.C.S. Eng., St. Bartholomew's Hospital. Physiology: J. Barlow, F.R.C.S. Eng., St. Mungo's College, Glasgow; C. H. Golding-Bird, M.B.Lond., F.R.C.S.Eng., Guy's Hospital; B. T. Lowne, F.R.C.S.Eng., Middlesex Hospital; W. Stirling, M.D., C.M.Edin., Owens College, Manchester. *Final Examination*—Midwifery: W. Duncan, M.D.Brux., M.R.C.P. Lond., F.R.C.S.Eng., Middlesex Hospital; M. Handfield-Jones, M.D. Lond., M.R.C.P.Lond., M.R.C.S., St. Mary's Hospital; G. E. Herman, M.B.Lond., F.R.C.P.Lond., F.R.C.S.Eng., London Hospital; A. J. McC. Routh, M.D.Lond., M.R.C.P.Lond., M.R.C.S.Eng., Charing Cross Hospital. *Diploma in Public Health*—Part I.: G. Turner, M.B.Cantab, M.R.C.S.Eng., L.R.C.P. Lond., Guy's Hospital. Part II.: Sir G. Buchanan, M.D.Lond., F.R.C.P.Lond., London Fever Hospital. And Mr. H. Morris, F.R.C.S.,Eng., Middlesex Hospital, was elected a Member of the Court of Examiners for a period of five years.

The following gentlemen, having passed the necessary examinations, and having conformed to the By-laws and Regulations, have been admitted Fellows of the College:—G. A. Hamerton, M.D.Brux., L.R.C.P. Lond.; E. Clarke, M.D.Lond.; A. W. Cadman, L.K.Q.C.P.I.; P. W. M. Howse, L.R.C.P.Lond.; C. D. Green, M.D.Lond.; J. Griffith, L.R.C.P.Lond.; F. H. Westmacott, L.R.C.P.Lond.; R. M. Littler, L.R.C.P.Lond.; A. E. Mahood, M.B.R.U.I.; J. M. Hall, M.D.R U.I.; R. G. Hogarth, L.R.C.P.Lond.; T. G. Ouston, L.R.C.P.Lond.; W. P. Purvis, M.B.Lond., L.R.C.P.Lond.; H. L. Rutter, M.D.Durh., L.R.C.P. Lond.; H. W. Armstead, M.B.Lond., L.R.C.P.Lond.; G. T. S. Sichel, L.R.C.P.Lond.; E. Henry, L.R.C.P.Lond.; G. D. E. Jones, L.R C.P. Lond.; T. D. Lister, L.R.C.P.Lond.; W. L. Christie, M.D.New Zealand; F. Beben, M.B.Cantab., L.R.C.P.Lond.; T. H. Boyd, M.B.Melb.; J. S. Buchanan, M.B.Glasg., L.R.C.P.Lond.; G. Wilkinson, M.B. Cantab., L.R.C.P.Lond.; H. B. Grimsdale, M.B.Cantab., L.R.C.P. Lond.; A Keith, M.D.Aberd.; J. B. Leathes, M.B.Oxon., L.R.C.P. Lond.; G. O. Rigby, M.B.Melb.; F. Barrington, M.B,Edin. Seven other candidates passed the examination, but not having yet complied with the By-laws, will receive their diplomas at future meetings of the Council, and twenty-three candidates were referred.

The following gentlemen having previously passed the necessary examinations, and having now attained the legal age of 25 years, were admitted Fellows of the College:—E. W. Adams, L.R.C.P.Lond.; A. N. Weir, L.R.C.P.Lond.; F. B. Rutter, M.B.Durh., L.R.C.P.Lond. The following gentleman having passed the necessary examinations was admitted a member of the College:—A. Delve, L.S.A., University College Hospital.

